# Letter on “Protection by exclusion? The (lack of) inclusion of adults who lack capacity to consent to research in clinical trials in the UK”

**DOI:** 10.1186/s13063-020-4054-4

**Published:** 2020-01-21

**Authors:** Sarah Griffiths, Lorna Manger, Rebecca Chapman, Lauren Weston, Ian Sherriff, Cath Quinn, Paul Clarkson, Caroline Sutcliffe, Karen Davies, Richard Byng

**Affiliations:** 1 University of Plymouth, Plymouth, UK; 2 Dementia Partnerships Lead, University of Plymouth, Plymouth, UK; 3 Senior Lecturer in Social Care and Society, University of Manchester, Manchester, UK; 4 University of Manchester, Manchester, UK; 5 D-PACT Chief Investigator, University of Plymouth, Plymouth, UK

Dear editors,

We read with interest the recent article regarding the lack of inclusion of people who lack capacity in trials: ‘Protection by exclusion? The (lack of) inclusion of adults who lack capacity to consent in clinical trials in the UK’ [1]. As the authors so eloquently spotlight, those without capacity in many population groups are those who are likely to be most vulnerable to frailty, comorbidities and isolation. Their exclusion from research significantly limits the specific evidence base for their care. As a research team developing and evaluating a Dementia Support Worker intervention based in primary care, we appreciate the challenges of including people in complex dementia interventions research who are most likely to benefit from the intervention. We are mindful of one of five ‘Dementia statements’, developed by people living with dementia and grounded in Human Rights law: ‘We have a right to know about and decide if we want to be involved in research that looks at cause, cure and care for dementia and be supported to take part’ [1].

We wished to develop an ethical recruitment process that enabled us to involve and understand the barriers to involvement of hard-to-reach groups of people with dementia: those who lack capacity but also those who lack support networks and experience frailty and emotional difficulties. We have developed a person-centred flexible recruitment pathway in consultation with our Patient and Public Involvement (PPI) group (including people with dementia and carers). The approach involves the practice team, Clinical Research Network (CRN), staff, and the research team working seamlessly together. The approach is pro-active, using a combination of letters, phone calls, invites to clinics and a home visit as appropriate and depending on the response at each stage. Many people with dementia do not necessarily read or understand letters, so we cannot assume that no response means engagement in decision-making about involvement. At the same time, we aim to avoid unwelcome pursuit; declining to take part results in no further contact, and involvement requires actively opting in.

A key principle of the Mental Capacity Act [3] is that individuals must be supported to understand information relevant to a decision, retain information, weigh up that information and communicate a decision by any means possible. Where people lack capacity, a personal consultee can be involved in decision-making based on prior knowledge of the person’s preferences and values. The Department of Health [2] provides guidance on nominating a consultee. This is not without challenges. Capacity can fluctuate over time. Also, a person’s personality, preferences and values may change as the dementia progresses. Those who may have been willing to engage in research may become more suspicious and unwilling, for instance. These dilemmas will need to be addressed on a case-by-case basis, but even if a consultee is involved, the role of verbal and non-verbal signs of assent from the person with dementia should not be ignored.

In order to try to address some of these challenges, our recruitment pathway allows ongoing assessment of the nature of family support and barriers to involvement, over time and prior to consent. Judgements about capacity and about who might reasonably act as consultee will result from this ongoing process and from developing an understanding of an individual’s communication abilities and needs, rather than relying on a test score on one day at one time. Crucially, if there is no response from an eligible participant at any of the stages of the pathway, a clinical home visit is built in. This will involve a clinical assessment first in case there are unknown needs.

Our journey through the ethics process has been challenging. Pre-trial development work for both intervention and trial procedures is not well understood. We were also very aware that there have been different views about whether CRN staff can access clinical records or make telephone calls prior to consent. The first panel we applied to gave an unfavourable opinion. As our intervention was not fully developed, it was advised that we should initially recruit only those with capacity. Of course, there are some research designs that require only participants with intact cognition, depending on the demands of participation, but to exclude those who lack capacity in our study would have been highly restricting in terms of the population we are aiming to support. Our PPI group expressed incomprehension at this stage: ‘It feels unethical to exclude them’ ‘How do you ever change things for people without capacity?’ The panel also said that only the practice team should approach individuals prior to consent, making the role of CRN staff redundant. A favourable opinion was given by a second panel, who recognised the process of complex intervention development and advised that we should recruit people who lack capacity. They identified an ethical imperative to do this. They also recognised that research network staff could be incorporated into an enhanced primary care team and so ensure that the process would not be affected by the primary care workforce crisis.

Shepherd et al. (2019) have provided a catalyst for increased awareness, debate and action in this important area. Through our feasibility phase, we are currently testing out our approach to recruitment and look forward to contributing to this debate by sharing what we learn from this process.

A wider challenge for the future will be around improving consistency of interpretation and advice from ethics panels so that hard-to-reach groups are enabled to take part in intervention development research.

Dementia Person Aligned Care Team (D-PACT) research team:

Sarah Griffiths (Research Fellow, University of Plymouth), Lorna Manger (Research Assistant, University of Plymouth), Rebecca Chapman (Assistant Trial Manager, University or Plymouth), Lauren Weston (Research Assistant, University of Plymouth), Ian Sherriff (Dementia Partnerships Lead, University of Plymouth), Cath Quinn (Senior Research Fellow, University of Plymouth), Paul Clarkson (Senior Fellow, National Institute for Health Research (NIHR) School for Social Care Research, University of Manchester), Caroline Sutcliffe (Research Fellow, University of Manchester), Karen Davies (Research Associate, University of Manchester), Richard Byng (D-PACT Chief Investigator, University of Plymouth).

Email: d-pact@plymouth.ac.uk. Twitter: @DementiaPACT. Website: https://www.plymouth.ac.uk/research/primarycare/dementia-person-aligned-care-team. Funding: NIHR programme grant: RP-PG-0217-20,004.

## Data Availability

Not applicable.

## Abbreviations

CRN: Clinical Research Network
PPI: Patient and Public Involvement
